# Oral health care beliefs among care personnel working with older people – follow‐up of oral care education provided by dental hygienists

**DOI:** 10.1111/idh.12588

**Published:** 2022-02-02

**Authors:** Kristina Edman, Inger Wårdh

**Affiliations:** ^1^ Oral and Maxillofacial Surgery Department of Surgical Sciences Medical Faculty Uppsala University Uppsala Sweden; ^2^ Center for Public Dental Services Falun Sweden; ^3^ Center for Clinical Research Uppsala University/Region Dalarna Falun Sweden; ^4^ Department of Dental Medicine and Academic Center for Geriatric Dentistry Karolinska Institute Stockholm Sweden; ^5^ Department of Health Sciences Karlstad University Karlstad Sweden

**Keywords:** care of older people, care personnel, education, nursing home, nursing OHCB, nursing staff, oral health care beliefs

## Abstract

**Objectives:**

The proportion of older people in the population is increasing rapidly. Along with this comes an increase in the number of people requiring assistance in daily living, including oral care. Swedish law stipulates that care personnel who work with older people should be offered oral health education every year. The aim of this study was to investigate oral health care beliefs among such personnel.

**Methods:**

A questionnaire study was conducted among 2167 personnel providing care to older people at special accommodation sites and in home care. Data were collected using the Nursing Dental Coping Beliefs Scale. Descriptive statistics were calculated and logistic regression analysis was performed.

**Results:**

Personnel working in home care had lower odds of having an internal locus of control than those working in special accommodation, and personnel with less than 10 years of working experience had lower odds than their more experienced counterparts. Men had higher odds of having an external locus of control than women.

**Conclusions:**

It seems important to ensure that home care personnel and less experienced personnel attend oral care educational sessions, and to encourage male staff to focus on oral care work.

## 1 INTRODUCTION

The proportion of older people in the population is increasing rapidly,^1^ and along with this comes an increase in the number of people requiring assistance in daily living. The consequences of increased dependency include loss of ability to perform oral care such as tooth brushing or interproximal cleaning without assistance, which carries a risk of impaired oral health. Oral health in developed countries has improved, and the proportion of older people retaining their natural teeth or fixed reconstructions has increased.^2^ It can be assumed that older people are therefore at an increased risk of oral health complications, and have an increased need for high‐quality oral care.

This improvement in oral health is a positive development, but also poses a challenge to nursing home personnel who provide care for older people needing support with daily oral health care. Studies investigating oral health among older people in short‐term care have found that toothlessness is rare but oral health is poor.^3^, ^4^ Maintaining oral health is vital for older people's general health, well‐being and quality of life.^5^, ^6^, ^7^, ^8^, ^9^, ^10^ Poor oral health has a negative impact on general health and quality of life in this population,^11^ and the number of remaining teeth is important for nutrition, swallowing function^12^ and good dietary habits.^13^ Although personnel who provide care to older people believe that good nursing also includes oral care,^14^, ^15^ this type of care is often neglected^16^ and has low priority in the daily work.^17^ Despite efforts to increase the oral health knowledge and oral care skills of nursing personnel, oral hygiene among older people living in special accommodation is often inadequate.^18^

A Swedish law which took effect in 1999 regulated both dental remuneration and dental care for dependent older people. The law states that these people should have access to a free‐of‐charge oral health care assessment in their residence, as well as subsidized basic dental care and, where applicable, nursing home personnel with training in oral health care.^19^ Regulations also stipulate that nursing home personnel should be offered three hours of basic education in oral disease and oral hygiene, with one hour of follow‐up education once a year. The basic education comprises theoretical information about oral hygiene and diseases such as dental caries, periodontitis, oral yeast infections and mucosal wounds, for example caused by dentures. Newly hired personnel generally do not receive their basic education immediately; but whenever a sufficient number of them have accumulated, the unit manager or the medically responsible nurse informs the dental team that there is now a reasonably sized group available for training. The follow‐up education includes updates on the information provided in the basic training, and is often customized on the basis of knowledge gaps expressed by the care personnel or identified by the dental team. All personnel actively involved in nursing of the residents (i.e. licensed nurses, assistant nurses and unit managers) are invited to participate in the basic and follow‐up education.

In Dalarna, a region in central Sweden, there are approximately 8000–8500 individuals working in special accommodation, home care and accommodation for disabled people. The public dental care service in Dalarna has a goal of reaching 40% of personnel in its provision of yearly follow‐up education. In 2020, only 1% of nursing home personnel participated in basic education and 7% in yearly follow‐up education. This low participation rate might be due to the Covid‐19 pandemic, which made it very difficult to offer education because of restrictions on physical meetings. In addition, nursing home personnel had a very high workload because of sick leave among their colleagues. The corresponding percentages in 2019 were 23% and 32%, respectively. Basic and follow‐up education is delivered by dental hygienists from the public dental service. Since the law changed in 1999, no evaluation has been made of the oral health education provided to nursing personnel. The aim of this study was therefore to explore the oral health care beliefs of care personnel working among older people.

## 2 MATERIALS AND METHODS

### 2.1 Design and sample

This questionnaire study included personnel in older care who were responsible for and actively involved in patient care; licensed nurses, assistant nurses, unit managers and others, such as nursing students actively involved in patient care, working both in the homes of older people and in sheltered accommodation in Dalarna, Sweden. Besides ethical approval, permission was obtained from the social managers in the different municipalities. A total of 4500 questionnaires were distributed.

### 2.2 Procedure

Dalarna is divided into 15 municipalities, both rural and urban, and contains 96 special accommodation sites and 69 home care groups employing approximately 4500 personnel. In June 2019, the study population were invited to complete a self‐administered questionnaire including both the Nursing Dental Coping Beliefs Scale (Nursing DCBS) and items covering background data such as age, gender, number of years of experience, education, position at work, form of employment and workplace. Supplementary questions about the importance of the resident's oral health as well as the participants’ own oral health were also asked. The unit manager distributed the questionnaires at a team meeting and collected them back in as soon as the participants had finished answering them. Personnel who were not able to attend the team meeting completed the questionnaire at another time and returned it to the unit manager. Participation was voluntary, and personnel gave their consent by completing the questionnaire.

### 2.3 Measurement

The Nursing DCBS is an instrument used to measure oral health care priority among nursing personnel. It was adapted from the original Dental Coping Beliefs Scale (DCBS),^20^ and has been tested for validity and reliability.^17^ The instrument comprises a 28‐item questionnaire covering four dimensions: internal locus of control (IL), external locus of control (EL), self‐efficacy (SE) and oral health care beliefs (OHCB). In its original form, the DCBS consists of a 44 item paper‐and‐pencil questionnaire concerning various beliefs about a person's control over oral health, and was developed in the United States to measure the effect of individual oral health care instruction.^20^, ^21^ Three models of cognitive psychology were used to generate and select items for the questionnaire: locus of control, self‐efficacy and the cognitive model of behavioural change. The scale was tested among male veterans as part of a larger oral hygiene study.^20^

#### 2.3.1 Locus of control

Locus of control is a concept in personality psychology, developed in the 1950s by American psychologist Julian Rotter to describe people's beliefs about their own degree of control over the events that occur in their lives. Having an *internal* locus of control means believing in the possibility of controlling one's own life, whereas an *external* locus of control reflects the belief that life is controlled by chance, fate, or external factors beyond one's control.^22^

#### 2.3.2 Self‐efficacy

The concept of self‐efficacy was originally proposed by another psychologist, Albert Bandura, as part of a theoretical framework for analysing and predicting changes achieved in fearful and avoidant behaviour following different forms of treatment. It describes an individual's belief in their own ability to successfully execute the behaviours required to produce particular outcomes.^23^

#### 2.3.3 Oral health care beliefs

The dimension of oral health care beliefs covers knowledge and beliefs about oral disease. It was outlined by Donald Meichenbaum with the purpose of identifying faulty beliefs or misconceptions.^24^ For the DCBS, this dimension was adjusted to suit health beliefs involving oral hygiene.^21^

### 2.4 Data analysis

The data were analysed using version 26.0 of IBM SPSS (SPSS Inc., Chicago, IL, USA). Descriptive analyses, frequencies and distributions were calculated. The five‐point Likert scale was trichotomized into ‘agree/completely agree’, ‘disagree/completely disagree’ and ‘no idea’. The items were grouped in the four dimensions covering SE, EL, IL and OHCB (seven in each dimension).^17^ A reliability test was performed using Cronbach's alpha. Sum scores were calculated and classified into maximum (score of 35 = 1) versus others (0) for the dimensions EL, IL and OHCB. Due to the low number of maximum responses for the dimension SE, we classified the score into high (score of 33 = 1) versus others (0). Logistic regression was used to analyse associations between dental coping beliefs and the participants’ years of experience, age, position at work (nurse/assistant nurse vs. unit manager/other, such as health care students), education (elementary school, secondary school or college of higher learning/university), form of employment (permanent vs. locum tenens) and workplace (home care vs. special accommodation). Significance level was set at *p* ≤ 0.05. A model of multiple logistic regression was tested but did not provide any added value for the study and was therefore not included.

This study was approved by the Swedish Ethical Review Authority (No: 2018/300) and was conducted in accordance with the international ethical principles established by the Declaration of Helsinki. The study was registered at ClinicalTrials.gov (ref: NCT03376022).

## 3 RESULTS

### 3.1 Background characteristics

Of the 2167 caregivers who completed the survey (48% response rate), 1933 were women and 199 were men; the remaining 35 participants did not answer the question on gender. Their mean age was 44.2 (SD: 12.6) years, and they had a mean of 17.6 (SD: 11.6) years of work experience. When divided by workplace, mean age was 45.2 (SD: 12.3) years among those in special accommodation and 42.9 (SD: 12.9) years among those in home care, and length of work experience was 19.4 (SD: 11.9) years and 15.3 (SD: 10.8) years, respectively. Basic characteristics of the participants are given in Table 1. The majority had permanent employment, and were assistant nurses. In the total group, 52% had received basic education in oral disease and oral hygiene and 43% had received follow‐up education.

**TABLE 1:** Basic characteristics of the participants

	n	%
Workplace*		
Home care	902	42
Special accommodation	1228	58
Education**		
Elementary school	264	13
Secondary school	1645	80
College/university	157	7
Position at work***		
Licensed nurse	17	1
Assistant nurse	1588	76
Unit manager	32	2
Other	443	21
Form of employment****		
Permanent	1887	90
Locum tenens	203	10
Basic education in oral health^5^		
Licensed nurse	8	47
Assistant nurse	885	57
Unit manager	16	52
Other	153	35
Follow‐up education/training in oral health		
Licensed nurse	8	47
Assistant nurse	739	47
Unit manager	20	62
Other	122	28

* n = 37

** n = 101

*** n = 87

**** n = 77

### 3.2 Nursing dental coping beliefs scale

Table 2 presents Cronbach's alpha for the four dimensions, ranging from 0.44 to 0.64. An attempt was made to improve the Cronbach's alpha values by excluding the lowest‐value items, but this did not result in a significant improvement and so the items were left unchanged. Table 2 also displays the results for the DCBS in the different dimensions.

**TABLE 2:** Percentage (%) of responses for all items, grouped in the four dimensions (n = 2167)

Item	Agree/completely agree (%)	Disagree/completely disagree (%)	No idea (%)
*Self‐efficacy (Cronbach's α 0.442)*			
I know how to prevent oral yeast infections	76.8	8.1	15.2
I know how oral mucosal wounds can be treated	34.1	32.4	33.6
I know how to use dental floss correctly	90.4	4.5	5.1
I can successfully remove the majority of plaque to prevent cavities and gum disease	40.5	35.3	24.2
Using toothbrush and dental floss correctly can prevent tooth problems	92.8	4.0	3.2
If I were given oral health care training, I would be better able to perform oral hygiene for older people	81.0	8.8	10.2
If I knew more about dental diseases, I would be able to practice better oral care for older people	79.0	9.2	11.8
*Internal locus of control (Cronbach's α 0.569)*			
Tooth cavitation can be prevented	88.8	7.8	3.4
Tooth brushing can help prevent tooth cavitation	97.0	2.0	1.0
Gum diseases can be prevented	82.2	6.7	11.1
I believe teeth can be preserved for a lifetime	57.2	33.3	9.5
I think dental floss can prevent tooth loss	77.9	8.2	13.9
I think older people desire help with oral hygiene	76.8	10.0	13.2
I think older people eat better with a healthy mouth	94.3	2.8	2.9
*External locus of control (Cronbach's α 0.642)*			
Tooth brushing and using dental floss do not help if your own parents had bad teeth	10.6	82.0	7.4
I think that tooth loss is normal when you are getting old	21.9	64.3	13.8
Even if you take good care of your teeth, they fall out as you get older	13.6	76.9	9.5
Only the dentist can prevent teeth cavitation and gum diseases	9.5	87.7	2.8
It is not possible to prevent sickness/medicines destroying teeth	42.7	36.5	20.8
One method of brushing is just as effective as any other	21.5	61.1	17.4
I think dentures can stay in the mouth even at night time	22.7	67.7	9.6
*Oral health care beliefs (Cronbach's α 0.544)*			
Visiting the dentist is only necessary when experiencing pain in the teeth	9.0	88.5	2.5
Once gum disease has started, it is almost impossible to stop it	19.4	43.5	37.1
If the gums bleed while tooth brushing, you should stop brushing	3.8	93.9	2.3
If the gums bleed while using dental floss, you should stop flossing	5.5	88.5	6.0
I think older people will say if they need help with oral hygiene	21.2	70.8	8.0
Use of fluoride is most suitable for children	12.3	72.3	15.4
I think dentures create fewer problems than natural teeth	32.2	50.3	17.5

#### 3.2.1 Self‐efficacy

The caregivers considered that correct use of a toothbrush and dental floss would lead to fewer tooth problems, and that oral health care training would improve their ability to perform oral hygiene care. The majority agreed on how to use dental floss correctly (90.4%) and how to prevent oral yeast infections (76.8%), but there was uncertainty over how to treat wounds in the oral mucosa and in trusting their ability to successfully remove plaque in order to prevent cavities and gum disease.

#### 3.2.2 Internal locus of control

The majority of the caregivers considered that oral disease could be prevented, and that tooth brushing and dental floss were of help with this (77.9%). They also believed that older people ate better with a healthy mouth (94.3%), and that these people wanted help with performing oral hygiene care (76.8%). There was some uncertainty regarding the belief that teeth could be preserved for a lifetime.

#### 3.2.3 External locus of control

Most of the caregivers (87.7%) did not consider that the dentist was the only one able to prevent teeth cavitation and gum diseases, or that brushing teeth and using dental floss was of no use if one's parents had had bad teeth (82.0%). Opinions were split regarding whether sickness and medicine destroyed teeth. The majority did not agree that the method of tooth brushing was irrelevant, and also did not agree that tooth loss was normal when aging.

#### 3.2.4 Oral health care beliefs

Most of the caregivers disagreed either partly or completely that gum disease was impossible to stop once started. However, a relatively large proportion was uncertain or did not know (37.1%). The majority were aware that it was not correct to stop flossing or brushing if gum bleeding occurred, and believed that older people would not tell their caretaker if they needed help with oral hygiene.

No differences were found between licensed nurses/unit managers and assistant nurses/others (data not shown).

### 3.3 Supplementary questions regarding the residents’ oral health and the care personnel's own oral health

Table 3 shows the results of supplementary questions. The participants considered both the residents’ and their own oral health to be very important. Over 60% of the participants did not perform oral cleaning if the older person refused.

**TABLE 3:** Percentage (%) of responses to supplementary questions (n = 2167)

Item	Agree/ completely agree n (%)	Disagree/completely disagree n (%)	No idea n (%)
I think the residents’ oral health is important	2110 (99)	‐	12 (1)
I perform oral care for the residents	1967 (88)	194 (9)	62 (3)
I think it is important to perform oral care for the residents	2095 (98)	14 (1)	21 (1)
If the older people refuse help I refrain from performing oral care	1318 (62)	568 (27)	231 (11)
My own oral health is important	2117 (99)	14 (1)	‐

Table 4 shows the relation between the four dimensions and background variables presented as odds ratio (95% CI). Compared with their younger counterparts, participants aged 50 and older had 8.88 times greater odds of having high OHCB (2.13–37.06). Staff working in home care had 0.57 times lower odds of having IL (0.36–0.88) than those in special accommodation, and the participants with more than 10 years of work experience had 2.45 times greater odds of having IL compared with those who had less experience (1.05–5.73) and men had 2.23 greater odds of external locus of control than women (1.14–4.36). No differences in SE or IL were found between those who had attended basic and/or follow‐up education in oral care and those who had not. Those who had attended basic oral care education had greater odds of having EL (OR: 2.63, 95% CI: 1.38–5.00) and OHCB (OR: 2.66, 95% CI: 1.40–5.05).

**TABLE 4:** Logistic regression models showing relations between the four dimensions and background variables presented as odds ratios (OR) with 95% confidence intervals (CI). Significant associations (*p *≤ 0.05) are highlighted in grey

	External locus of Control	Internal locus of control	Oral health care beliefs	Self‐efficacy
	OR (95% CI) P	OR (95% CI) P	OR (95% CI) P	OR (95% CI) P
*Workplace* Special accommodation (ref) Home care	1.31 (0.79, 2.17) 0.291	0.57 (0.36, 0.88) 0.012	0.94 (0.56, 1.56) 0.802	0.72 (0.44, 1.16) 0.171
*Position at work* Licensed nurse/assistant nurse (ref) Unit manager/other	1.08 (0.60, 1.95) 0.797	0.74 (0.43, 1.27) 0.277	0.86 (0.46, 1.59) 0.624	0.62 (0.33, 1.15) 0.131
*Education* Elementary school (ref) Secondary school College/University	0.85 (0.41, 1.75) 0.658 0.88 (0.29, 2.69) 0.827	1.76 (0.80, 3.85) 0.160 2.32 (0.86, 6.22) 0.096	1.06 (0.48, 2.38) 0.882 2.16 (0.78, 5.92) 0.136	1.64 (0.70, 3.83) 0.253 1.06 (0.29, 3.82) 0.929
*Basic oral care education*	2.63 (1.38, 5.00) 0.003	1.47 (0–91, 2.36) 0.112	2.66 (1.40, 5.05) 0.003	0.66 (0.09, 4.73) 0.683
*Follow up education in oral care*	1.52 (0.87, 2.67) 0.142	1.15 (0.75, 1.76) 0.516	1.55 (0.91, 2.65) 0.108	1.55 (0.26, 9.30) 0.631
*Form of employment* Permanent (ref) Locum tenens	0.46 (0.14, 1.49) 0.198	0.89 (0.42, 1.87) 0.759	0.30 (0.07, 1.24) 0.096	0.82 (0.35, 1.91) 0.643
*Working years* <5 years (ref) 5–10 years >10 years	0.41 (0.10, 1.74) 0.227 2.29 (0.91, 5.80) 0.080	1.77 (0.68, 4.63) 0.242 2.45 (1.05, 5.73) 0.038	0.45 (0.13, 1.62) 0.225 1.91 (0.81, 4.50) 0.138	0.58 (0.25, 1.31) 0.188 0.75 (0.40, 1.42) 0.382
*Age group* <30 years (ref) 31–50 years >50 years	1.64 (0.60, 4.44) 0.335 2.24 (0.85, 5.92) 0.102	0.99 (0.56, 1.76) 0.977 0.73 (0.40, 1.34) 0.311	3.78 (0.86, 16.56) 0.077 8.88 (2.13, 37.06) 0.003	0.68 (0.37, 1.28) 0.236 0.63 (0.33, 1.18) 0.149
*Gender* Female (ref) Male	2.23 (1.14, 4.36) 0.019	1.19 (0.61, 2.33) 0.617	0.69 (0.25, 1.93) 0.481	0.26 (0.06, 1.06) 0.061

## 4 DISCUSSION

In this study population, there was some uncertainty about taking care of the older person's oral hygiene, how to treat oral mucosal wounds and the impact of medicines on oral health. Furthermore, personnel working in special accommodation had greater odds of having an internal locus of control than personnel working in the older people's homes, and personnel with more than 10 years of working experience had greater odds than their less experienced counterparts. Men had greater odds of high external locus of control than women.

Of the 4500 distributed questionnaires, 2167 were returned and included in the study, and so the dropout rate was high (52%). Other similar studies have shown dropout rates ranging from 20% to 75%.^25^, ^26^, ^27^ The poor response rate may indicate that oral healthcare is considered low priority and perhaps not something of interest. The response rate was particularly low among licensed nurses and unit managers, who despite having ultimate responsibility for the residents’ nursing care, including oral health care, generally do not undertake oral care themselves. This might have influenced their ability to respond to the questionnaire, and must be taken into consideration when interpreting the results.

The DCBS index was developed, tested and validated among staff in nursing homes, and was found to be a suitable tool to use even in small nursing staff groups.^17^ Another study using the Nursing DCBS^28^ tried to improve the internal consistency by excluding certain items from the index, which produced Cronbach's alpha values of 0.542–0.711. In our study we made similar attempts by excluding the lowest‐value items, but this did not result in a significant improvement. The low alpha values (0.442–0.642) can be seen as an indication that the instrument was not optimal for use in this population of different professions with varying degrees of medical education, working in special accommodation and in home care.

Knowledge of the importance of oral health and how to achieve it has historically been poor among personnel who provide care to older people.^29^ The oral health of older people has improved despite this, but there are still indications that knowledge remains inadequate.^27^, ^30^ Care personnel need to become more confident in providing oral care to older people, and better oral care training is needed.^31^ The personnel in this study considered oral health to be important – their own as well as that of the residents – and appeared to know the importance of oral hygiene in preventing tooth problems, indicating a high self‐efficacy. However, as other studies have found,^31^, ^32^ the vast majority expressed a need for oral health care training as well as theoretical teaching about oral disease in order to be able to provide better oral care and to treat mucosal wounds.

Internal locus of control, comprising the belief that one's own actions and control are of importance, was high; the only uncertainty was over whether teeth could be preserved for a lifetime. This might have been due to deficient knowledge of dental diseases and treatment strategies. The actions of the staff might also have been hindered by resistant behaviour in residents, which is common.^33^ In our study, over 60% of the participants refrained from oral health care if the resident was uncooperative, a barrier highlighted in other studies.^15^, ^34^ It can be assumed that in a stressful work environment where many tasks are to be carried out, the residents’ oral hygiene is not a high priority and staff are unwilling to argue with the residents.

The personnel working in home care were less aware of items concerning preventive care than those working in special accommodation. A possible explanation is that the home care personnel were younger and had less working experience, as well as more varying nursing educational training. Furthermore, older people living at home are expected to be able to take care of their own oral hygiene themselves, which can affect the personnel's interest and sense of importance of oral care. It can also be assumed that staff turnover is more frequent in home care services than in special accommodation, and that the dental team which provides oral health education had failed to reach older care personnel in home care. Previous studies have found that care personnel with longer experience have a more positive attitude to oral care,^29^ which is consistent with our study. This could be explained by the personnel's own experiences of the implications and consequences of poor oral health.

Scores varied on external locus of control, comprising the belief that control is located outside the person's own ability to influence, or that events are controlled by chance or fate. Items referring to oral hygiene were considered to be under the individual's control, while factors such as ageing and sickness, and their impact on oral health, were not. Deficient knowledge and faulty beliefs regarding oral disease were identified in some aspects of oral health care beliefs; specifically, concerning whether dentures cause fewer problems than natural teeth and whether it is possible to stop gum disease once it has started. Most of the aspects were comparable with other studies,^29^, ^35^ and a relationship with age could be seen. Beliefs about oral hygiene and oral care may partly depend on educational level^33^ and cultural differences.^35^ The participants’ own experiences of oral disease, unsuccessful treatment or dental fear might have influenced their answers as well as their general attitudes to oral health.

There is a need for implementation of oral care routines early in the care planning, and documentation of these routines in the residents’ records.^25^, ^36^, ^37^, ^38^ It has also been emphasized that the complex healthcare situation of older people means that theoretical education for care personnel is not sufficient, and that individual hands‐on guidance may be a better approach,^39^, ^40^ as seen too in this study.

Only about half of the care personnel in our study reported that they had participated in the basic and follow‐up education offered by the dental care system. The Dalarna public dental care service did not reach its goal of offering yearly follow‐up education to 40% of older care personnel in either 2019 or 2020, though in 2020, this was likely due to the Covid‐19 pandemic that resulted in restrictions on gatherings. The unit manager has the responsibility for ensuring that all care personnel are given the opportunity to participate in theoretical oral health education. Practical hands‐on training is supposed to be given to the personnel when the dental team visit the residents for oral health assessment, but this is not always possible due to working schedules, high workload, stress and lack of time.^33^

Lack of oral health education in the basic education curriculum for nurses and assistant nurses might have influenced both the willingness of staff to participate in this kind of investigation and the answers given by the participants in this study. It might affect care personnel's perceptions of the importance of oral health and care, which could explain some of our results.

In future research, one possible strategy would be to consult with communities in planning an educational programme that better suits their needs and working conditions, and to follow up on the training and educational activities provided. Other beneficial actions would be to make the basic and follow‐up education provided by dental care mandatory for all care personnel, and to point out the need for an overview of the formal course content regarding oral health in the basic education programmes in health and older care. It would also be beneficial to include oral health in the individual care planning.

## 5 CONCLUSIONS

Based on our results, it seems important to ensure that home care, and less experienced personnel attend oral care educational sessions, and to encourage male staff to focus on oral care work.

## 6 CLINICAL RELEVANCE

### 6.1 Scientific rationale for the study

To improve oral health care beliefs in care personnel, there is a need for an overview of current oral health care education.

### 6.2 Principal findings

Oral health care education has to be highlighted, specifically amongst less experienced older care personnel and home care personnel. There is a need to improve male staff members’ locus of control concerning oral health care.

### 6.3 Practical implications

This study demonstrates the importance of mandatory basic and follow‐up education provided by dental care for all care personnel, and points out the need for an overview of the formal course content regarding oral health in the basic education programmes in health and older care.

## CONFLICTS OF INTEREST

The authors declare no conflict of interest. The funder had no role in the design of the study; in the collection, analysis or interpretation of data; in the writing of the manuscript; or in the decision to publish the results.

## AUTHOR CONTRIBUTIONS

Conceptualization, K.E; methodology, K.E and I.W; software, K.E; validation, K.E and I.W; formal analysis, K.E; investigation, K.E.; resources, K.E; data curation, K.E; writing – original draft preparation, K.E and I.W; writing – review and editing, K.E and I.W; visualization, K.E; project administration, K.E; funding acquisition, K.E. All authors have read and agreed to the published version of the manuscript.
